# Pet Food Quality Assurance and Safety and Quality Assurance Survey within the Costa Rican Pet Food Industry

**DOI:** 10.3390/ani9110980

**Published:** 2019-11-15

**Authors:** Astrid Leiva, Andrea Molina, Mauricio Redondo-Solano, Graciela Artavia, Lizeth Rojas-Bogantes, Fabio Granados-Chinchilla

**Affiliations:** 1 Centro de Investigación en Nutrición Animal (CINA), Universidad de Costa Rica, Ciudad Universitaria Rodrigo Facio, 11501-2060 San José, Costa Rica; andrea.molina@ucr.ac.cr (A.M.); fabio.granados@ucr.ac.cr (F.G.-C.); 2 Escuela de Zootecnia, Universidad de Costa Rica, Ciudad Universitaria Rodrigo Facio, 11501-2060 San José, Costa Rica; 3 Centro de Investigación en Enfermedades Tropicales (CIET) and Facultad de Microbiología, Universidad de Costa Rica, Ciudad Universitaria Rodrigo, 11501-2060 San José, Costa Rica; mauricio.redondosolano@ucr.ac.cr; 4 Centro Nacional de Ciencia y Tecnología de Alimentos, Universidad de Costa Rica, Ciudad Universitaria Rodrigo Facio, 11501-2060 San José, Costa Rica; graciela.artavia@ucr.ac.cr (G.A.); lizeth.rojasbogantes@ucr.ac.cr (L.R.-B.)

**Keywords:** quality assurance, safety, pet food, good manufacturing practices

## Abstract

**Simple Summary:**

The health status of pets may be affected by infectious agents transmitted through the feed. The close coexistence between humans and animals makes the safety assurance of pet food paramount. Different unit operations are applied at the industry level to prevent and reduce the presence of physical, chemical, and biological hazards in pet food; through these operations and adequate implementation of good manufacturing practices, the safety of the finished product can be preserved.

**Abstract:**

Costa Rican animal feed production is continually growing, with approximately 1,238,243 metric tons produced in 2018. Production-wise, pet cat and dog food are in fifth place (about 41,635 metric tons per year) amongst animal feeds, and it supplies up to 90% of the national market. Pet food production has increased as a response to the increase in the population of dogs and cats in Costa Rica, where 50.5% of households own at least one dog and indicates more responsible ownership in terms of feeding pets. Part of the process of making dry pet food involves a thermal process called extrusion, which is capable of eliminating the microbial load. However, extrusion can compromise nutritional quality to some extent by denaturing proteins, oxidizing lipids, and reducing digestibility. The objective of this study was to evaluate the quality and safety of dry pet food and to assess the effect of the extrusion process on digestibility and the quality of proteins, amino acids, and fatty acids. Pet food samples were collected before and after extrusion and were used to evaluate Good Manufacturing Practices (GMP), based on Central American Technical Regulation (RTCA 65.05.63:11). In general terms, weaknesses in infrastructure, documentary evidence, and post-process practices were observed in two Costa Rican feed manufactories. Feed safety was surveyed through the analysis of *Salmonella* spp., *Escherichia coli*, *Listeria* spp., *Staphylococcus aureus*, aerobic mesophilic microorganisms, fungi, and yeasts counts. The extrusion process effectively reduced pathogenic microorganisms, and showed no effect on the digestibility of dog food (*p* = 0.347), however, it could reduce the availability of some nutrients (e.g., amino acids, fatty acids). Furthermore, a retrospective diagnosis was made for puppy food (*n* = 68), dog food (*n* = 158), and cat food (*n* = 25), to evaluate the history of nutritional quality and safety. Finally, it can be confirmed that the correct implementation of GMP allows feed manufacturers to deliver a product of optimum texture, smell, nutritional composition, and safety.

## 1. Introduction

The pet population in Costa Rica was reported in 2018 to be 400,000 cats and 1,400,000 dogs [1], where 50.5% of households are home to at least one dog. Pets play a particularly important role in the lives of people, including the emotional and physical wellbeing of their owners [2,3] who nowadays consider their pets as “family members” [3]. Appropriate nutrition is essential to satisfy the needs of pets effectively, ensuring their good health and long-term well-being [4,5,6]. As pet owners have become more aware of the quality of food, they feed their pet companions; commercial dry pet food has become a staple [4]. For example, 76.5% of Costa Rican pet owners feed domestic animals balanced/complete pet foods [7], which indicates a growing trend in responsible pet ownership. 

The estimated production of the Costa Rican balanced-animal-feed industry is 1,238,243 metric tons per year, with 41,635 tons per year used for cat and dog food, the fifth most significant category in the national production scheme [1]. Furthermore, from a total of 129 feed mills, 37 are pet food facilities [1]; such figures are not negligible for a country like Costa Rica. Given the close relationship between animal feed and food for human consumption, and based on a comprehensive production system under the slogan “from farm to fork” [8], health, safety, and quality standards to ensure feed and food safety are increasingly stringent [9]. In the food industry, good manufacturing practices (GMP) are the basis for reducing the risk of physical, chemical, and biological hazards entering at any point in the food production chain [10].

During dry pet food production, conditioning and extrusion processes are performed at temperatures ranging from 80 to 150 °C, which have the advantage of reducing waste, increasing the shelf life of the product, and improving the digestibility of carbohydrates [11,12,13]. However, during extrusion, vitamin activity may be reduced, lipids can suffer oxidative degradation, and amino acid availability may be reduced [14]. Hence, optimal and standardized processing conditions must be maintained to minimize adverse effects on the final product [14,15]. Additionally, the temperatures reached during the extrusion process eliminate pathogenic bacteria, such as *Salmonella* spp. that could be found in the food [16]. On the other hand, food drying and cooling processes reduce the moisture content to a maximum of 13 g/100 g and the temperature to approximately 20 °C, respectively, providing the final product with optimum conditions for storage and inhibition of bacterial and fungal growth [12,17].

Dry pet foods represent an economical alternative for feeding pets; since they are easily stored [4], and manipulated by pet owners, and, as a balanced feed, they should provide all the nutritional needs of the animal with few relatively small servings. Hence, it is necessary to evaluate both the value of the pet food in the market and the food system as a whole [5]. Herein, we report several assays to assess quality in the puppy, dog, and cat food. This report presents data on the evaluation of GMP in two Costa Rican pet food-manufacturing facilities, with a particular focus on microbiological safety through the elaboration process. 

## 2. Materials and Methods 

### 2.1. Analyzed Samples

Sampling was carried out at different stages of the process (one area after the mixing process and another at the end of the processing line) within two pet food production establishments located in Costa Rica. A minimum of two samples of each production batch (i.e., four samples per batch—two after mixing and two of the finished product) were sampled at least five times per establishment. Sampling was performed according to the procedure described in the Association of American Feed Control Officials (AAFCO) [18].

Our analysis included dry extruded dog food (*n* = 15), dry extruded puppy food (*n* = 10), and dry extruded cat food (*n* = 5). The number of samples was determined by a retrospective analysis and was proportional to the volume of the different types of pet foods found in the market (i.e., dog > puppy > cat food). Samples consisted of commercially available presentations of pet foods (i.e., finished product samples). All samples were quartered and sieved to 1 mm particle size following AOAC 950.02. 

The number of samples was determined based on a retrospective analysis of the data from the 2012–2018 surveillance program. The analysis considered the most common feedstuffs used in Costa Rica, target growth stage, import and export regulations, contamination risk, the productivity of the pet food industry, and the risk for human and animal health associated with each food type. For example, some analyses were included during the retrospective analysis of puppy food that are not comprised in the adult food analyses, as young dogs may be more susceptible to certain nutrient deficiencies (i.e., iron AOAC 968.08/975.03, selenium AOAC 996.17, gross energy US ISO 9831:1998, sugars [19]). 

### 2.2. Reagents and Quality Control Materials 

For quality control purposes, proficiency test samples 201825 (dry cat food) and 201923 (dog food) from the AAFCO’s check sample program were used for all chemical/nutritional assays, including fatty acid, amino acid profiling, and water activity. Sample 201754 (dog food) from the quarterly mineral scheme was used as quality control for calcium, phosphorus, and heavy metal analysis.

### 2.3. Evaluation of Good Manufacturing Practices (GMP)

A GMP diagnosis was made based on the Central American Technical Regulation (RTCA 65.05.63:11, see Appendix A).

### 2.4. Food Safety: Microbiological Analysis

As part of the microbial evaluation of the samples collected, *Salmonella* spp., total coliform bacteria, *Escherichia coli*, and the enterohemorrhagic *E. coli* O157:H7 analyses were performed. Assays were based on ISO 17025 accredited methods AOAC 967.25/967.28/994.04/978.24 [20], and APHA/CMMEF methods 9.91–9.94 (based on an MPN technique) and 34.22–34.24, respectively. *Listeria* spp. and *Staphylococcus aureus* in feed were detected, analyzed, and confirmed following the methodology by Nemser and collaborators [21], and Rodríguez-Lázaro and collaborators [22].

### 2.5. Food Safety: Heavy Metal Analysis 

The heavy metal analysis was performed using a methodology described by Granados-Chinchilla and coworkers [23]. Briefly, 300 mg of the sample was placed into the digestion vessel (DAP-60+, Berghof Products + Instruments GmbH, Baden-Wurtemberg, Eningen, Germany) and was mixed with 10.0 mL of HNO_3_ (65 mL/100 mL, puriss, Sigma-Aldrich 84378, St. Louis, MO, USA). The mixture was microwave-digested using a 3 step temperature program. Step 1: 175, 30, 5, 5, 70, step 2: 230, 30, 1, 10, 90, step 3: 50, 25, 1, 10, 0 (temperature [°C], pressure [bar], heating time [min], hold time [min], power [%]). Limit of detection and quantification for arsenic, cadmium, mercury, and lead in dog food were 1.00 and 3.03, 0.01 and 0.03, 0.02 and 0.06, and 0.60 and 1.82 μg kg^−1^, respectively. 

### 2.6. Nutritional Quality: Proximate Analysis

Dry matter (loss on drying/moisture), crude protein, fiber, and ash, as well as calcium, phosphorus, and pepsin digestibility of animal protein assays, were performed to assess the nutritional quality of the pet food. All tests were performed using ISO 17025 accredited methods based on AOAC 930.15, 988.05/984.13/976.06/990.02/990.03, 962.09, 942.05, 968.08/975.03/985.35, 965.17/986.24, 935.13, and 971.09, respectively. Crude fat and water activity (a_w_) were calculated in extruded pet foods by acid hydrolysis AOAC OMA^SM^ 954.02, and Aqualab chilled mirror methods (measurement performed at 24.50 ± 0.24 °C, Aqualab 4TE, Decagon Devices, Pullman, WA, USA), respectively. 

### 2.7. Nutritional Quality: Fatty Acid Profiling 

A 250 g pet food sample was milled and sieved to 1 mm (using a ZM 200 ultracentrifuge mill, Retsch GmbH, Haan, Germany). After that, a subsample of ca. 1 g of each feed sample was placed in a 50 mL glass beaker, where 5 mL of diethyl ether was added and mixed using an ultrasonic shaker (USC200TH, VWR International, Center Valley, PA, USA) for 5 minutes. Afterward, a 200 µL aliquot was transferred to a GC 2 mL vial (Agilent Technologies, Santa Clara, CA, USA). Then, 800 µL of diethyl ether and 1000 µL of, a previously prepared, 0.25 g/100 mL TMHA solution in methanol were added to the same vial. An aliquot of 2 µL of the resulting mixture was injected into the GC system. Qualitative analyses of the volatile compounds were carried out using an Agilent gas chromatograph (7820, Agilent Technologies, Santa Clara, CA, USA) equipped with an Agilent Technologies J&W DBWAX microbore column of 10 m length, 0.1 mm diameter, 0.1 µm film thickness and Agilent 5977E mass spectrometer (MSD). The carrier gas was helium at a constant flow rate of 0.3 mL min^−1^. The GC oven temperature was kept at 50 °C for 0.34 min and increased to 200 °C at a rate of 72.51 °C min^−1^. The temperature was kept at 200 °C constant for 0.17 min, increased to 230 °C at a rate of 8.7 °C min^−1^, and remained constant for 7.9 min for a total run time of 13.93 min. The split ratio was adjusted at 30:1. The injector, transfer line, ion source, and quadrupole temperatures were set at 250, 250, 230, and 150 °C, respectively. The mass range and the electron energy was set at 50–450 m/z and 70 eV, respectively. Constituents were identified by matching their spectra with those in NIST library 14. Only hits with a match factor above 80% were considered. FAME mixtures GLC-486 (*n* = 40 analytes) and GLC-860 (*n* = 60 analytes, Nu-Chek Prep, Inc., Elysian, MN, USA) were used as quality control comparing retention times and mass spectra with those found in the analyzed samples. Compounds used to check mass tuning included tetradecanoic (6.16 min; [M+] 227.6 m/z), pentadecanoic (6.72 min; [M+] 243.4 m/z), hexadecenoic (7.58 min; [M+] 256.3 m/z), octadecanoic (9.70 min; [M+] 285.5 m/z), cis-13-octadecanoic (10.21min; [M+] 285.7 m/z), 9Z-octadecenoic (7.78 min; [M+] 284.1 m/z), and (Z,Z)-9,12-octadecadienoic (10.86 min; [M+] 280.0 m/z) acids. Enanthic acid (≥99%, 75190, Sigma-Aldrich, St. Louis, MO, USA) was used as an internal standard and 9c11t-C_18:2_, 10c12t-C_18:2_, C_12:0_, 4c7c10c13c16c19c-C_22:6_, 11t-C_18:1_ were concurrently monitored by simultaneous ion monitoring (SIM) mode (total dwell time 100 ms and cycles 8.3 Hz. For compounds with no standard, identification should be considered as tentative.

### 2.8. Nutritional Quality: Amino Acid Profiling and Furosine Analysis

Sample digestion was carried out as previously reported for furosine [24]. Briefly, feed samples were sieved to 1 mm, and 200 ± 1 mg subsamples were used for digestion. The sample was transferred into a 40 mL glass vial (27184 SUPELCO, St. Louis, MO, USA), mixed with 2 mL water (to achieve dispersion), and 6 mL of an HCl 10.6 mol L^−1^ aqueous solution. Immediately, the vial was capped with a septum (tan PTFE/silicone, 27188-U, SUPELCO, St. Louis, MO, USA), and nitrogen was bubbled for 1 min into the solution through a needle. The resulting mixture was heated adiabatically for 23 h at 110 °C. The resulting hydrolysate was filtered by gravity through a grade 4 qualitative filter paper (Whatman, GE Healthcare Life Sciences Pittsburgh, PA, USA). Then, 130 µL of a borate buffer (Merck Millipore, Burlington, Massachusetts, USA, B0394, 50 mmol L^−1^, pH = 10), 10 µL sample digest, 20 µL NaOH (Merck Millipore, 1064679010, 3.6 mol L^−1^), 10 µL OPA freshly prepared solution (Merck Millipore, P0657, 10 mg *o*-phtalaldehyde is dissolved in 500 µL chromatographic grade ethanol and made up to volume with borate buffer in a 10 mL volumetric flask), 10 µL FMOC (Merck Millipore, 160512, 10 mg 9-fluorenylmethoxycarbonyl chloride is dissolved in 500 µL chromatographic grade acetonitrile and made up in borate buffer in a 10 mL volumetric flask), 320 µL ultrapure water were sequentially mixed together in a HPLC ready-to-inject vial. One microliter of the resulting mixture was injected into the LC system. The solvent system consisted of a 40 mmol L^−1^ NaH_2_PO_4_ buffer adjusted at 7.8 pH (Merck Millipore, S9638, ACS, 98% pure, solvent A) and acetonitrile, methanol, and water (45:45:10, solvent B). Gradient mode was as follows: 0% B at 0 min, 0%B at 1.9 min, 57% B at 18.1 min, 100% B at 18.6 min, 100% B at 22.3 min, 0% B at 23.2 min, and 0% B at 26 min. Chromatographic separations were performed using an Agilent Technologies LC system equipped high resolution column (150 × 4.6 mm, 3.5 µm, PN 963400–901260), a 1260 infinity quaternary pump (61311C), column compartment (G1316A), an automatic liquid sampler module (ALS, G7129A) and a fluorescence detector (G1321A) (Agilent Technologies, Santa Clara, CA, USA). A detection system was set at 340 (excitation) and 450 nm (emission) for all amino acids except proline for which 266 and 305 nm wavelengths were used, respectively. Ultrapure water [type I, 0.055 µS cm^−1^ at 25 °C, 5 µg L^−1^ TOC] was obtained using an A10 Milli-Q Advantage system and an Elix 35 system (Merck KgaA, Darmstadt, Germany). The identity of each amino acid was assessed, and each compound quantified using NIST^®^ SRM^®^ 2389a amino acids in 0.1 mol L^−1^.

### 2.9. Statistical Analysis 

Descriptive statistics were performed on the GMP data for each facility. The same data set was used to detail the prevalence of each of the microorganisms, and the average digestibility of the product, by sampling area and by the facility. The total number of coliforms, aerobic mesophilic microorganisms, and fungal count in the finished product analyzed using a One-Way ANOVA Tukey’s honestly significant difference *post hoc* test. The same analysis was used for the digestibility of the product before and after going through the extrusion process. A value of α = 0.05 was used as the significance level for all hypothesis tests. InfoStat (version 12, Córdoba, Argentina) was used to conduct all statistical analyses.

## 3. Results and Discussion

### 3.1. GMP

**Figure 1:**
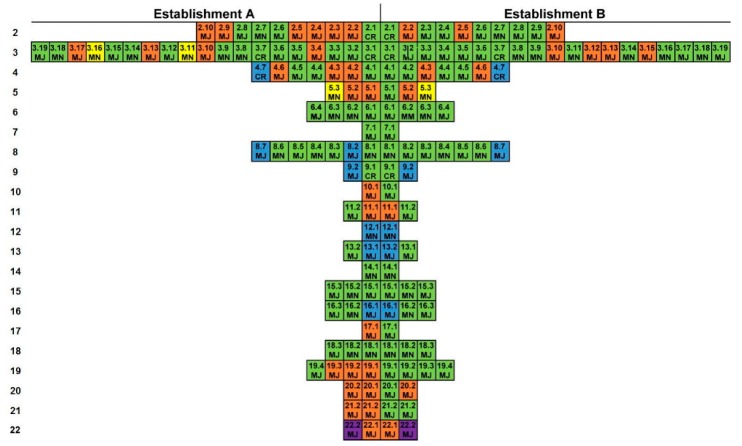
Critical point evaluation for two pet-food-manufacturing facilities. Each box denotes a different evaluation point from the technical regulation (RTCA (Central American Technical Regulation) 65.05.63:11). The evaluation points were as follows: (2) Documentation; (3) facilities; (4) equipment; (5) personnel; (6) pest control; (7) production flow; (8) raw materials; (9) storage of risk ingredients; (10) water; (11) formulation; (12) grinding; (13) adding ingredients; (14) mixing; (15) packaging and labeling; (16) storage; (17) reprocessing; (18) dispatch, distribution, and transportation; (19) quality and safety controls; (20) traceability; (21) verification of GMP; and (22) environment. Non-conformities were classified as CR: Critical; MJ: Major; and MN: Minor in terms of severity of the risk they pose. Color scale denotes 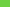 conformity; 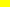 minor non-conformity; 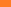 major non-conformity; 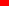 critical non-conformity; 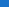 evaluation point not applicable; 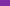 point not evaluated.

The results of the evaluation carried out on each feed mill are summarized in Figure 1. A color code was used to identify specific points with nonconformities (NC). 

For Table A1 (2. Documentation), none of the facilities complied with points 2.2, 2.5, and 2.10. In facility A, the GMP manual is outdated, and in establishment B, it has not yet been enforced. Then, establishment A breaches point 2.3 and 2.4. 

**Figure 2:**
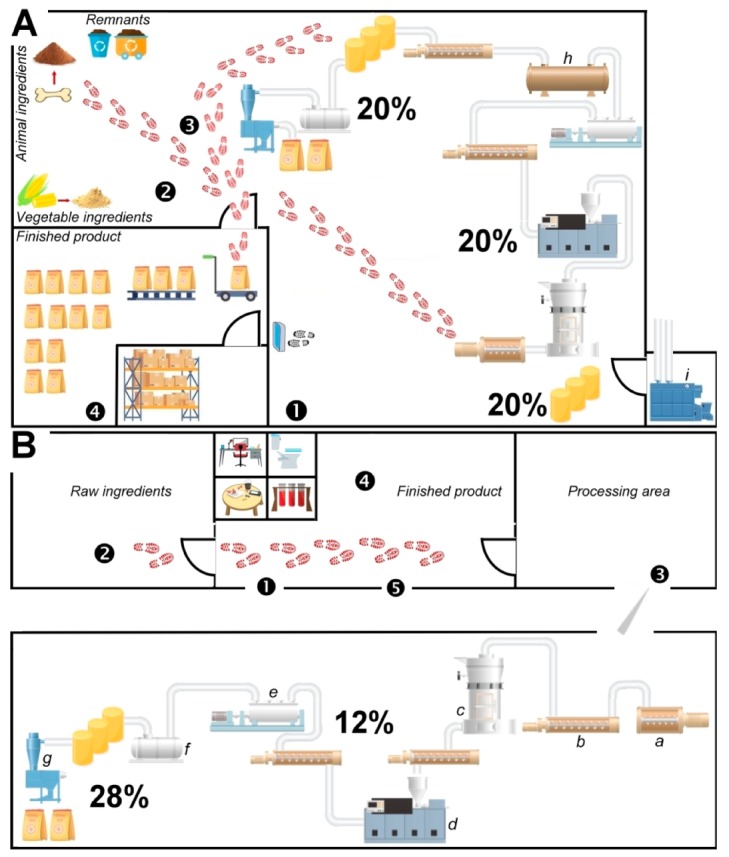
Representation of pet food production facilities that were assessed during the survey. Establishment (**A**) makes several extruded feeds, including pet food and (**B**) solely purposed to pet food. In sequence, (**1**) Raw material input; (**2**) Raw material storage; (**3**) Processing areas; (**4**) Finished product storage; (**5**) Finished product output.Red footprints indicate inadequate transit between the finished product and raw material transport within work areas. (**a**) Mixer; (**b**) screw conveyor; (**c**) mill; (**d**) conditioner and extruder; (**e**) dryer; (**f**) oil-sprayer; (**g**) sieve and packer; (**h**) cooler; (**i**) boiler. Percentages represent the relative number of samples tested for this production point. Black outlines represent physical barriers among areas.

In Table A1 (3. Facilities), which evaluates the facilities, both establishments do not follow points 3.10 and 3.13. Infrastructure in both facilities is old and has not been remodeled in recent years. Windows and doors allow the entrance of birds and other animals, which increases the risk of contamination of raw materials and the finished product. The first defense against biological hazards in a production system relies on the facilities [25]; therefore, facilities represent a critical point. Moreover, neither facility had a protocol for people or external vehicles access, nor there was a clear written indication for the correct flow of workers and visitors through the feed plant. Additionally, establishment A exhibited non-conformities for points 3.4, 3.11, 31.6, and 3.17. The first point relates to the lack of a vehicle access procedure. The second point is associated with the infrastructure condition; and the last two with the lack of written procedures and records that function as evidence of the activities that are carried out. For example, garbage dumps (a source of pollution) are not well identified, and the frequency of waste disposal is not stated, as it should be [10]. Establishment B failed to comply with points 3.12 and 3.15. Failing to comply with 3.12 may indicate a risk of cross-contamination in the finished product (Figure 2A,B). In the case of point 3.15, the design of the facilities does not allow for effective cleaning. A diagram of the design of each of the facilities and process flows is represented (Figure 2A,B). In facility B, walls separate each area of the food plant, however, the process flow should be corrected as the transit lines between the raw materials and the finished product area should never cross (red steps, Figure 2A,B). Any contamination, especially biological hazards, can occur through employee traffic or the relocation of equipment [25]. 

In Table A1 (4. Equipment), both facilities breach points 4.3 and 4.6, sensitive issues. None of the facilities showed evidence that their equipment guarantees accuracy [26] or that batch-to-batch cross-contamination does not occur [25,27]. Establishment A also has NC in 4.2, where it failed to collect evidence that shows magnets and screens are routinely checked, a critical point, in physical hazards avoidance [10,25].

In Table A1 (5. Personnel), facility A failed to comply with the three points that make up this tier, while establishment B, only failed points 5.2 and 5.3. The collaborating staff, being directly in contact with the food, must ensure their health status and their training in food handling [26,28].

Table A1 (6. Pest control) and Table A1 (7. Production flow) are fully compliant, and these sections are decisive to maintain the safety of the final product. However, it is difficult to evaluate the pest control system in practice and the production flow beyond the design. On the other hand, pest control should include the management of birds, that are recognized as carriers of *Salmonella* spp. and insects [27].

In Table A1 (8. Raw materials) and Table A1 (9. Storage of risk ingredients), no NC was found. In Table A1 (10. Water), facility A, presented an NC in 10.1, since their water quality analyses are outdated. In Table A1 (11. Formulation), both establishments failed to comply with 11.1, due to a lack of evidence to show that the staff verifies formulas. Table A1 (12. Grinding) is not applicable (NA) for either of the facilities. Table A1 (13. Addition of ingredients), Table A1 (14. Mixing), Table A1 (15. Packaging and labeling), and Table A1 (16. Storage) did not have NC. In Table A1 (17. Reprocessing), on reprocesses, facility A breached the only point in the section. Table A1 (18. Dispatch, distribution, and transportation), did not present NC. 

Table A1 (19. Quality and safety controls), facility A presented NC in 19.1, 19.2, and 19.3, that is, raw materials are used without evaluation of suppliers. Furthermore, a procedure for entering and handling raw materials is not available. The issue above represents another critical point since the reception of raw materials is an entrance for physical, chemical, and biological hazards [27]. Though keeping control of each batch of raw material may be taxing, an inspection of suppliers, considering the requirements of the ingredients to be acquired, will reduce potential risks [25]. On the other hand, it is not guaranteed that the finished product possesses the nutritional formulation that was previously calculated, and there are no routine safety controls. Therefore, there are no records of the complete product analysis, which is a violation of the confidence of the consumer on what is stated in the product label [10], and breach of official regulation [29]. 

In Table A1 (20. Traceability), facility A breaches the two points, 20.1 and 20.2, while establishment B only breaches 20.2; that is, there are no procedures or records to keep traceability. The procedures and logs allow controlling the process of production with quality and safety standards that enable its commercialization [29,30] and are an essential part of a GMP system. 

Facility A failed to comply with Table A1 (21. Verification of GMP), since they do not have an internal audit program (21.1), and are not executing the recommendations issued by the official authority (21.2). There is a non-compliance of Table A1 (22. Environment) since there is no clear procedure for handling solid and liquid waste (22.1). 

### 3.2. Nutritional Quality: Crude Protein and Digestibility before and after the Extrusion Process

Pet feeding is aimed to provide a better quality of life and to maintain optimal health status. Therefore, each serving offered to a cat or dog must ensure to cover the basic nutritional requirements based on its physiological stage and physical activity [31,32]. In the formulation of animal feed, both the quality of the raw materials and the manufacturing process are involved in the nutritional quality of the finished product [32,33,34]. Since 95% of dog food is extruded, it is characterized mainly by its low moisture content (i.e., below 10 g/100 g) [35], hardness, and durability [36].

As stated before, extrusion transforms, mix, and sterilizes a wide variety of ingredients, to produce a harmless, stable, and balanced food [34,37]. The same process that renders safe food can denature proteins, which consequently affects amino acid bioavailability [37]. It can also cause lipid oxidation, which may decrease the content of essential fatty acids, such as linoleic and linolenic acid [37]. 

Since not all nutrients are harnessed similarly, a standard indicator of food quality is digestibility, which is, measuring the proportion of nutrients available for absorption [38]. Table 1 shows the data obtained from the analysis of pepsin digestibility, moisture, crude protein, and fat performed on adult dog food samples. Moisture continually changes during the process and reduced to less than 10 g/100 g, one of the goals of the extrusion (Table 1) [36]. Crude protein values are below recommended may be associated with errors during formulation. Crude fat also varies, and, in some cases, it increases after exiting the extruder as flavoring and fat are added in a post-extrusion step (Table 1). Finally, no significant differences for protein digestibility are observed between the sampling areas, especially before and after the thermal process, which coincides with the results obtained by van Rooijen and collaborators [37], and Tran and collaborators [34] (Table 1). On the other hand, digestibility values equal to or greater than 80 g/100 g are recommended, since absorption by the animal is prominently improved for these values [35,38,39]. None of the feed samples tested met this requirement, which is related to the quality of raw materials [32] (Table 1).

### 3.3. Nutritional Quality: Water Activity and Moisture in Pet Foods

Water activity obtained for a dry extruded adult dog, puppy, and cat foods were 0.5356 ± 0.0961, 0.5837 ± 0.0682, and 0.5477 ± 0.0505, respectively (Table 2). These values are relatively higher than those reported elsewhere for cat food (0.30–0.50) and dog food (0.30–0.54) [40]. As a cost management strategy, Costa Rican feed industry usually maintains moisture contents between 8 and 10 g/100 g (Table 2). Increased water activity may have a severe impact on the pet food shelf life [41]. Water activity has demonstrated to be a functional alternative to moisture content analysis [42] as a_w_ is related to lipid quality and has proved to influence lipid oxidation [43], lipid modification [44], mycoflora, and fumonisin B_1_ accumulation [45].

### 3.4. Nutritional Quality: Crude Protein, Digestibility and Amino Acid Profile

Some feed ingredients used commonly in pet food formulations have been described elsewhere [46,47]. In Costa Rica, the basis for pet food formulations is corn and soybean meal. Additionally, fish, poultry by-products, meat and bone meals (i.e., swine and cattle and other ruminants) are regularly used to supply protein and fat, which is especially practical for animals that are strictly carnivore [6]. Our data for crude protein and amino acid profiles are in agreement with other reports [48]. Taurine is a dietary essential sulfur-containing amino acid for felines [49,50,51,52]. From the diets tested, *n* = 1 sample was below the taurine limits recommended for adult cats, which is of great concern as the endogenous synthesis of this compound by cats is limited and putative precursors (cysteic and cysteinesulfinic acids) cannot substitute its presence [51] (Table 3). Crude protein values were above the recommended threshold except for *n* = 1 and *n* = 3 samples of puppy and adult dog food (Table 3). Although no recommendation is given by AAFCO in regard of taurine for dogs, recent reports have established a connection among cardiomyopathy in golden retrievers, fed with commercial diets, and taurine deficiency [53]. Hence, average values of 0.17 ± 0.03 g taurine/100 g sample found during our survey maybe not enough for certain dog breeds; the amount that must be, then, more strictly monitored (Table 3). However, Tjernsbekk and coworkers [54] have already demonstrated that crude protein alone cannot be used to assess nutritional and protein quality, where digestibility and amino acid profile is crucial. Values below thresholds can be explained by the effect that the extrusion process has on the feed ingredients, including their protein quality and amino acid profile [55,56,57,58]. Fortunately, no puppy food samples were below AAFCO suggested thresholds. Mean values of apparent protein digestibility (75.68 ± 7.75 g/100 g protein using in vitro method) seem to be in accordance with those reported elsewhere [59] for puppy food, albeit on the low range of that stated (Table 3). However, this is not the case for adult dry dog food, where mean values (68.61 ± 3.76 g/100 g protein) are below those declared therein [59] (Table 3).

### 3.5. Nutritional Quality: Furosine Content in Pet Foods

Recently, cell lines have been reported to be sensitive to furosine, and that this compound reduced weight gain, and affected the functions of liver and kidney in animals [60]. Besides being widely used as a marker of thermal treatment and nutritional quality of food, furosine detection may have now safety implications. As dry pet food is extruded to improve the digestibility of nutrients and increase shelf life and food safety [14], pet food ingredients can suffer considerable thermal processing, such as the case of soy. Nevertheless, extrusion tends to increase furosine content in soy. At least two research groups have evaluated furosine generation in soybean-based feed products obtained under severe thermal treatment conditions and reported values of 66.55 ± 0.37 and 108.01 ± 8.97 mg/100 g protein [61,62,63]. Our data (Table 3) is in agreement with the results by Chiang [63,64] in dry dog food stored for 12 weeks at 37.8 °C. Increased concentrations of furosine are undesirable in pet food as they indicate impaired lysine utilization by the animal and compromised weight gain [65]. 

### 3.6. Nutritional Quality: Fatty Acids, Fiber, and Minerals (Ca and P)

Fat provides energy, twice as much as proteins and carbohydrates, to intervene in the health and good condition of the skin and fur, and increases the palatability of food [35]. Although fat is not an essential nutrient, it must meet the requirements of essential fatty acids [35]. Fatty acids, such as linoleic acid, are precursors of other compounds, such as arachidonic acid, eicosapentaenoic acid (EPA) or docosahexaenoic acid (DHA), in puppies. Said molecules are essential for the proper development of the nervous system, and their deficiency is associated with vision problems and learning problems [39] (Table 4).

Calcium and phosphorus are essential for bone development and are minerals vital throughout the life of the animal, especially during the growth phase to avoid rickets [35]. In addition, pet food should maintain calcium within the recommended minimum and maximum values, as well as the 2:1 (Ca:P) ratio. Inverting this ratio may result in inadequate calcium absorption [35,39]. 

Fiber, in food for dogs and cats, has a mechanical function, by intervening in the formation of the bolus and collaborating with mineral absorption and biliary metabolism [35,39] (Table 5 and Table 6).

### 3.7. Food Safety: Microbiological Quality 

Our microbiological survey included the sampling of strategic zones within each feed plant (i.e., after the mixer, the extruder, and finished product) (Table 7, Figure 2A,B). Sampling after the thermal process demonstrates its effectiveness in eliminating microorganisms (Table 7, Figure 2A,B). Sampling the finished product shows that the GMP implemented can maintain microbiological safety (Table 7, Figure 2A,B). However, testing the finished product alone is not an effective way to determine the absence of a microorganism. Routine control should include the critical stages in the production process [27]. Table 7 describes the number of samples per sampling area, in addition to the results for different microbiological analyzes. The conformity criterion was based on current international regulation, where the sternest limits have been taken as the threshold value. 

Raw materials, and those of animal origin, in particular, carry a microbial load that was detected in the first stage of the production chain. EFSA [27], and Huss and collaborators [32] indicated that animal-based ingredients are potential sources of *Salmonella* spp. for compound feed, a fact which was also experimentally demonstrated by Leiva and collaborators [70]. The second production stage reflects the effectiveness of the thermal process to eliminate microorganisms [10,70]. During dry dog food production, the extrusion process requires high temperatures (from 80 to 150 °C) for at least two minutes to achieve the destruction of 10^3^ CFU per 100 grams [25]. However, the eradication of the initial microbiological load does not exclude the possibility of subsequent contamination [25,70]. Our results indicated the finished product might have considerable mesophilic aerobic bacteria, fungi, and yeast counts, although not pathogenic, are indicators of quality and storage conditions [71].

*Salmonella* spp. and *E. coli* are pathogenic bacteria that can be transmitted to humans, either by contact with the animal or contaminated food [41]. The latter is relevant since dogs have a close relationship with humans [27,32]. The presence of pathogenic microorganisms in feed is evidence of a deviation from GMP, such as those mentioned above [10,28,32]. Once a biological hazard has entered the production chain, due to a failure in the GMP system, it is a challenge to eradicate it. Consequently, more expensive processes within the production are needed to reduce or eliminate the risk [25]. On the other hand, the total of the samples assayed were negative for both *Listeria* spp. and *S. aureus*.

### 3.8. Food Safety: Heavy Metal Contaminants

Mean values for lead (508.52 ± 305.72 and 703.01 ± 705.40 mg kg^−1^ for puppy and adult dog food, respectively) were consistently higher than other heavy metals (Table 8). These values are low when compared with previous reports of cadmium and lead in dog food in concentrations of 0.20 ± 0.01 and 3.23 ± 0.08 mg kg^−1^, respectively [72]. Low amounts of these contaminants are expected, as Costa Rica is a country without a mining industry; therefore, heavy metal contamination in the compound feed may come from mineral sources [23,73]. However, dogs have been documented to be resistant to relatively elevated dietary doses of lead (i.e., 10 mg kg^−1^; [74]). Diets with doses of heavy metals as the ones reported herein are safe for chronic consumption by pets (Table 8). Recently, red meat-based dog diets have been reported to contain higher values of lead [74]. Finally, pet food can be considered safe, and hazard notifications caused by arsenic and mercury, although scarce, have triggered stern actions, such as product recalls [75]. 

### 3.9. Retrospective Nutritional and Safety Analysis of Pet Foods

#### 3.9.1. Puppy Food

In the case of puppy food, the average value for crude protein (i.e., 23.70 g/100 g) is close to the recommended threshold of 22.50 g/100 g, which means that at least some of the food samples have crude protein below this point (Table 9). For example, the minimum value found for this type of food is 9.24 g/100 g, which is considered extremely low (Table 9). This result may be a consequence of pet treat mislabeling as compound food, when the actual formulation round 12 g crude protein/100 g. Low protein values lead to growth retardation [76]. In the case of minerals, calcium, phosphorus, and selenium were also found at values as low as 0.22, 0.48 g/100 g, and 0.21 mg kg^−1^, respectively (Table 9). An opposite trend was observed in crude fat and iron supplementation (Table 9). In the case of microbiological analysis, only *n* = 1/68 samples (1.47% prevalence) were found to be above the legal threshold for *Salmonella* spp. (Table 9). As the thermal processing of extruded compound feed was found to be adequate, it is expected that contamination was acquired, afterward, by inadequate handling of the finished food or cross-contamination from high-risk raw materials previous to or during packaging (e.g., meat and bone meal) [20,70]. Similar studies have not reported *Salmonella* on dry extruded dog food [21,77]. Noteworthy, similar prevalence values as those stated herein have been reported in industrialized countries, where most of the *Salmonella* strains were recovered from the processing equipment [78]. However, an outstanding 71.43% of samples were positive for total coliforms, a general indicator of hygiene [20] (Table 9). Comparable results have been found in commercially available dog food sold in bulk and sealed packages [77]. The authors noted that 43.8% of the samples had mesophilic microorganisms in the range from 100 to 10,000 CFU g^−1^, and 77% of the samples had mold and yeast counts from 0 to 100 CFU g^−1^. Our data showed a low microorganism load (i.e., 382 ± 340 CFU g^−1^, and 62.80 ± 47.57 MPN g^−1^ for the total mesophilic bacterial count and total coliforms, respectively) (Table 9). In the current study, bulk feed samples (a practice in our country is a called “feed quartering and repacking”) were not included, however, that market should be regulated since bulk sales can reduce food safety [77]. Interestingly, even when yeast and mold counts are relatively low, the presence of mycotoxins could indicate previous fungal colonization of the raw materials before formulation and thermal treatment. For example, aflatoxin B_1_, deoxynivalenol and fumonisin B_1_ were found in several samples, though aflatoxin and deoxynivalenol levels were below their respective threshold for pet food (i.e., 20 and 2000 μg kg^−1^) (Table 9) [20]. Fumonisin B_1_ had an average of 5.54 mg kg^−1^, a contamination level above the recommended guideline for the sum of fumonisin B_1_ and B_2_ of 5000 μg kg^−1^ [20]. On the other hand, contamination of pet foods with life-threatening pathogens (e.g., *Listeria* spp., *Salmonella* spp., toxigenic *E. coli* strains) has been reported only in raw pet foods in a collaborative study from the US [21]. 

#### 3.9.2. Adult Dog Food

Regarding crude protein and fat, the results are similar to was found in puppy food. On the contrary, Ca and P were at recommended values; however, the Ca/P ratio in some cases exceed the maximum recommended of 2.5 (Table 10). Calcium and phosphorus intake should be strictly monitored in dogs as malnutrition is a risk factor for developmental orthopedic diseases and secondary nutritional hyperparathyroidism in large breed puppies and dogs [79,80]. Finally, *n* = 2/158 samples (1.26%) were found to be contaminated with *Salmonella* spp. (Table 10). *Salmonella* outbreaks associated with dry pet food and treats have become a concern for these products since they serve as a means of pathogen exposure for pets and their owners [81]. 

#### 3.9.3. Cat Food

In the case of cat food, some samples were found to be below the recommended dietary minimum for both crude protein and fat (set at 26.0 and 9.0 g/100 g, respectively) (Table 4). Interestingly, all samples were compliant for calcium and phosphorus (Table 11). Similarly to puppy and dog food, *n* = 1/25 sample (4.00% prevalence) was found to be contaminated with *Salmonella* spp. (Table 11). In all the cases, the samples with low nutrient values may not be able to supply the needs for maintenance or growth. Thus, the addition of dietary supplements or an increase in the amount of food intake may be required [3].

## 4. Conclusions

Good manufacturing practices are the basis of food safety, and their correct implementation is the foundation for risk management. The thermal process to which dry dog food is subjected to is useful in the elimination of pathogens. The prevalence of microorganisms, whether pathogens or indicators in the finished food, is associated with cross-contamination and deviance from the GMP program. Shortcomings hinder safe food production and seem to be reflected mostly in the lack of documentation, precise procedures that engender incorrect practices, and incorrect workflow within the manufacturing plant. The quality of the infrastructure, access to the facilities, the untethered transit of people and vehicles, the handling of raw materials and the finished product, pest control, and cleaning procedures are critical points to consider when product safety is to be guarded. The data also supports the fact that cross-contamination of feed is an issue. The thermal process to improve the safety of pet foods is of no use if the final product will be contaminated downstream. Although the digestibility of food is not affected by the extrusion process, it can reduce the availability of some nutrients (e.g., amino acids, fatty acids). Formulations should account for the loss of nutrients during thermal treatment since some of these nutrients are present in low concentrations and are prone to lability. Additionally, the monitoring of some compounds that are being produced during the thermal process (e.g., Maillard reaction) may be required as they can dictate the quality of the pet food. Costa Rican pet foods seem to be safe regarding heavy metals, though these analytes should be included in quality routine analysis. 

## Figures and Tables

**Table 1:** Proximate analysis for samples per zone obtained from two pet food plants.

Sampling Zone	Establishment (*n*)	Moisture	Crude Protein	Crude Fat	Pepsin Digestibility
		Concentration g/100 g			
After mixing	A (5)	11.45	16.71	8.43	69.77
	B (0)	ND	ND	ND	ND
	Median	11.45	16.71	8.43	69.77 ^a^
After extruder	A (5)	12.67	16.49	8.29	70.85
	B (3)	17.81	13.46	7.55	65.60
	Median	14.87	15.19	7.97	68.60 ^a^
Finished product	A (5)	7.71	16.23	12.87	71.17
	B (7)	10.47	13.48	12.38	72.36
	Median	9.32	14.62	12.59	71.86 ^a^
Reference		Recommended values in the finished product			
[35,39]		10.00 (max)	18.00 (min)	5.50 (min)	80.00 (min)

ND: Not done. Values with same letter code do not vary significantly: ^a^ (*p* = 0.347).

**Table 2:** Dry matter content and water activity as nutritional quality parameters of selected commercial dry pet foods.

Nutrient/Parameter	Mean ± SD	Median	Maximum *	Minimum *
Puppy food (*n* = 9)				
Moisture, g/100 g	8.03 ± 1.17	8.50	9.51 [10.00]	6.17
a_w_	0.584 ± 0.068	0.597	0.669 [0.60]	0.434
Adult dog food (*n* = 15)				
Moisture, g/100 g	7.66 ± 1.46	7.52	9.91 [10.00]	5.39
a_w_	0.536 ± 0.096	0.549	0.679 [0.60]	0.372
Cat food (*n* = 5)				
Moisture, g/100 g	7.94 ± 0.87	7.80	9.48 [10.00]	6.86
a_w_	0.548 ± 0.050	0.533	0.637 [0.60]	0.499

* Values in square brackets represent concentrations recommended by AAFCO [39] and FDA [41] for each animal species and nutrient.

**Table 3:** Protein characterization as nutritional quality parameters of selected commercial dry pet foods.

Nutrient/Parameter	Mean ± SD	Median	Maximum *	Minimum *
Puppy food (*n* = 9)				
Crude protein, g/100 g	27.35 ± 3.61	27.23	32.89	20.85 [22.50]
Non-essential amino acids, g/100 g				
Alanine	1.30 ± 0.24	1.26	1.68	0.99
Aspartate	2.12 ± 0.29	2.15	2.65	1.55
Glutamate	4.69 ± 0.89	4.68	6.38	3.00
Glycine	1.35 ± 0.22	1.31	1.70	0.92
Serine	2.16 ± 0.30	2.10	2.59	1.63
Tyrosine	0.81 ± 0.22	0.79	1.11	0.44
Essential amino acids, g/100 g				
Arginine	1.71 ± 0.27	1.77	2.17	1.35 [1.00]
Histidine	0.73 ± 0.12	0.70	0.95	0.61 [0.44]
Isoleucine	0.82 ± 0.19	0.89	1.09	0.51 [0.71]
Leucine	2.98 ± 0.64	3.21	3.62	1.79 [1.29]
Lysine	1.18 ± 0.28	1.14	1.58	0.79 [0.90]
Methionine	0.48 ± 0.10	0.49	0.59	0.30 [0.35]
Methionine-cystine	1.26 ± 0.16	1.20	1.55	1.08 [0.53]
Phenylalanine	1.15 ± 0.20	1.20	1.42	0.86 [0.83]
Phenylalanine-tyrosine	1.99 ± 0.15	1.97	2.24	1.79 [1.30]
Threonine	1.39 ± 0.28	1.30	2.12	1.13 [1.04]
Valine	1.02 ± 0.23	1.04	1.42	0.86 [0.68]
Conditionally essential amino acids, g/100 g				
Cysteine/Cystine	0.75 ± 0.18	0.72	1.09	0.60
Proline	1.34 ± 0.38	1.34	2.11	0.58
Taurine	0.17 ± 0.03	0.19	0.22	0.12
Pepsin digestibility, g/100 g protein	75.68 ± 7.75	76.95	84.78	64.03
Furosine, mg/100 g	34.70 ± 10.07	28.80	53	24.50
Adult dog food (*n* = 15)				
Crude protein, g/100 g	20.45 ± 3.30	19.78	27.46	15.46 [18.00]
Non-essential amino acids, g/100 g				
Alanine	1.01 ± 0.32	1.05	1.46	0.39
Aspartate	2.55 ± 0.72	2.63	3.50	1.08
Glutamate	5.91 ± 0.96	5.75	7.88	4.17
Glycine	1.60 ± 0.77	1.32	3.75	0.96
Serine	1.00 ± 0.62	0.86	2.12	0.28
Tyrosine	1.07 ± 0.40	0.98	1.92	0.54
Essential amino acids, g/100 g				
Arginine	1.35 ± 0.56	1.33	2.32	0.27 [0.51]
Histidine	1.24 ± 0.37	1.20	1.97	0.68 [0.19]
Isoleucine	0.83 ± 0.14	0.79	1.09	0.63 [0.38]
Leucine	3.16 ± 0.27	3.19	3.71	2.71 [0.68]
Lysine	1.12 ± 0.52	1.06	2.39	0.15 [0.63]
Methionine	0.47 ± 0.17	0.45	0.87	0.29 [0.33]
Methionine-cystine	1.36 ± 0.23	1.39	1.72	1.08 [0.65]
Phenylalanine	0.94 ± 0.21	0.79	1.09	0.63 [0.45]
Phenylalanine-tyrosine	1.98 ± 0.52	2.03	2.97	1.10 [0.74]
Threonine	0.85 ± 0.36	0.91	1.59	0.28 [0.48]
Valine	0.89 ± 0.29	0.88	1.27	0.58 [0.49]
Conditionally essential amino acids, g/100 g				
Cysteine/Cystine	0.90 ± 0.20	0.86	1.27	0.58
Proline	1.41 ± 0.30	1.43	2.11	0.66
Taurine	0.17 ± 0.03	0.17	0.25	0.12
Pepsin digestibility, g/100 g protein	68.61 ± 3.76	67.45	80.25	65.32
Furosine, mg/100 g	27.80 ± 5.26	26.55	37.30	20.50
Cat food (*n* = 5)				
Crude protein, g/100 g	30.05 ± 3.41	28.91	36.69	27.00 [20.00]
Non-essential amino acids, g/100 g				
Alanine	1.51 ± 0.26	1.66	1.76	1.17
Aspartate	2.30 ± 0.24	2.33	2.65	2.01
Glutamate	6.15 ± 0.89	6.31	6.90	4.44
Glycine	2.02 ± 0.33	2.09	2.37	1.48
Serine	1.62 ± 0.83	1.53	2.68	0.63
Tyrosine	1.00 ± 0.10	1.01	1.12	0.88
Essential amino acids, g/100 g				
Arginine	1.50 ± 0.30	1.43	1.94	1.08 [1.04]
Histidine	0.72 ± 0.23	0.68	1.14	0.49 [0.31]
Isoleucine	1.59 ± 0.31	1.46	2.16	1.26 [0.52]
Leucine	3.12 ± 0.21	3.18	3.30	2.71 [1.24]
Lysine	1.32 ± 0.21	1.33	1.66	1.08 [0.83]
Methionine	0.58 ± 0.08	0.60	0.67 [1.50]	0.44 [0.20]
Methionine-cystine	1.00 ± 0.32	1.20	1.33	0.59 [0.40]
Phenylalanine	1.32 ± 0.26	1.35	1.61	1.01 [0.42]
Phenylalanine-tyrosine	1.92 ± 0.45	1.89	2.62	1.35 [1.53]
Threonine	1.21 ± 0.09	1.20	1.38	1.11 [0.73]
Valine	1.15 ± 0.26	1.66	1.76	1.17 [0.62]
Conditionally essential amino acids, g/100 g				
Cysteine/Cystine	0.69 ± 0.05	0.67	0.76	0.64
Proline	1.24 ± 0.13	1.30	1.35	1.00
Taurine	0.22 ± 0.08	0.22	0.24	0.19 [0.20]
Furosine, mg/100 g	34.38 ± 7.58	31.70	47.10	26.60

* Values in square brackets represent concentrations recommended by AAFCO [39] for each animal species and nutrient.

**Table 4:** Fat characterization as nutritional quality parameters of selected commercial dry pet foods.

Nutrient/Parameter	Mean ± SD	Median	Maximum *	Minimum *
Puppy food (*n* = 9)				
Crude fat, g/100 g	13.24 ± 1.41	12.69	15.37	11.68 [8.50]
Fatty acid profile, g kg^−1^				
Ethanedioic acid	20.27 ± 6.79	20.95	28.70	10.40
Propanoic acid	14.46 ± 9.25	12.30	29.30	2.00
Butanedioic acid	35.06 ± 33.70	24.20	109.40	7.40
2-methyl-hexanoic acid	14.23 ± 10.47	10.30	35.50	5.10
2-methyl-pentanoic acid	45.97 ± 47.34	26.40	111.20	0.30
Octanoic acid	10.24 ± 6.35	12.50	18.60	2.80
Undecanoic acid	16.90 ± 8.07	17.30	27.90	5.10
(*Z*)-9-Dodecenoic acid	28.10 ± 14.60	19.00	48.70	16.60
Tetradecanoic acid	19.95 ± 2.79	20.90	22.60	15.40
Hexadecanoic acid	378.27 ± 89.09	410.00	511.20	257.00
Octadecanoic acid	78.11 ± 23.37	81.80	105.60	40.00
(*Z*)-9-Octadecenoic acid	212.61 ± 37.89	206.80	309.90	173.70
(*Z*,*Z*)-9,12-Octadecadienoic acid	112.99 ± 32.99	115.10	173.80	48.70 [13.00]
(*Z*)-13-Octadecenoic acid	214.90 ± 46.30	214.90	261.20	168.60
SFA	590.88 ± 122.96	568.80	813.10	348.90
MUFA	291.30 ± 61.62	276.70	396.90	183.00
PUFA	132.51 ± 80.36	116.30	254.10	3.90
Adult dog food (*n* = 15)				
Crude fat	12.52 ± 1.55	12.59	15.56	10.60
Fatty acid profile, g kg^−1^				
Ethanedioic acid	16.33 ± 10.66	13.10	33.80	1.40
Propanoic acid	11.90 ± 12.60	5.50	33.20	0.50
Butanedioic acid	15.18 ± 11.31	10.65	36.60	1.80
Pentanoic acid	3.68 ± 4.52	1.90	15.50	1.10
Dodecanoic acid	20.77 ± 14.82	15.80	46.70	5.90
Tetradecanoic acid	15.70 ± 8.61	15.55	29.30	2.20
Hexadecanoic acid	359.10 ± 94.31	341.00	547.00	224.90
(*Z*)-9-Hexadecenoic acid	12.63 ± 1.62	13.30	14.20	10.40
Octadecanoic acid	76.24 ± 37.49	76.50	169.80	22.40
(*Z*)-9-Octadecenoic acid	283.57 ± 45.81	291.30	352.20	192.70
(*Z*,*Z*)-9,12-Octadecadienoic acid	194.88 ± 54.65	186.70	328.00	122.50
(*Z*,*Z*,*Z*)-9,12,15-Octadecatrienoic acid	7.43 ± 2.19	7.65	10.10	4.30 [0.80]
(*Z*,*Z*,*Z*)-11,14,17-Eicosatrienoic acid	8.98 ± 4.05	10.45	12.80	2.20
SFA	526.79 ± 151.31	545.90	810.60	290.70
MUFA	317.74 ± 84.48	315.35	495.30	169.70
PUFA	167.40 ± 90.98	207.40	345.30	19.60
Cat food (*n* = 5)				
Crude fat, g/100 g	12.60 ± 1.57	12.94	14.43	10.74
Fatty acid profile, g kg^−1^				
Ethanedioic acid	19.48 ± 18.09	11.70	48.50	0.80
Propanedioic acid	12.68 ± 8.51	12.85	24.40	0.80
Butanedioic acid	17.51 ± 9.54	19.80	31.70	3.40
Butanoic acid	8.58 ± 7.66	8.10	17.90	0.20
2-methyl-decanoic acid	13.30 ± 4.36	12.20	19.10	8.60
Undecanoic acid	8.50 ± 1.90	8.50	10.40	6.60
Tetradecanoic acid	48.10 ± 16.78	47.45	71.30	26.20
Hexadecanoic acid	368.38 ± 61.73	352.30	460.60	298.00
(*Z*)-9-Hexadecenoic acid	29.10 ± 7.35	26.90	39.00	21.40
Octadecanoic acid	97.33 ± 14.70	106.40	109.00	76.60
(*Z*)-9-Octadecenoic acid	192.13 ± 89.01	212.95	289.50	36.40
(*Z*,*Z*)-9,12-Octadecadienoic acid	128.70 ± 67.41	128.70	227.00	44.70 [6.00]
(*Z*)-10-Tetradecen-1-ol acetate	114.50 ± 84.40	114.50	198.90	30.10
SFA	560.92 ± 147.65	559.40	842.90	391.80
MUFA	287.85 ± 119.73	326.40	387.80	42.40
PUFA	151.35 ± 53.10	137.30	236.30	78.90

* Values in square brackets represent concentrations recommended by AAFCO [39] for each animal species and nutrient.

**Table 5:** Fiber content as nutritional quality parameters of selected commercial dry pet foods.

Nutrient/Parameter	Mean ± SD	Median	Maximum *	Minimum *
Puppy food (*n* = 9)				
Crude fiber, g/100 g	2.35 ± 0.61	2.27	3.11	1.40
Adult dog food (*n* = 15)				
Crude fiber, g/100 g	2.76 ± 0.58	2.67	3.78 [4.00]	1.90
Cat food (*n* = 5)				
Crude fiber, g/100 g	1.98 ± 0.53	1.73	2.98	1.51

* Values in square brackets represent concentrations recommended by AAFCO [39] for each animal species and nutrient.

**Table 6:** Macro mineral profile as nutritional quality parameters of selected commercial dry pet foods.

Nutrient/Parameter	Mean ± SD	Median	Maximum *	Minimum *
Puppy food (*n* = 9)				
Calcium, g/100 g	1.57 ± 0.44	1.66	2.13 [1.80]	0.97 [1.20]
Phosphorus, g/100 g	1.04 ± 0.21	0.95	1.39	0.82 [1.00]
Ca/P ratio	1.49 ± 0.26	1.53	1.89 [2:1]	1.18 [1:1]
Adult dog food (*n* = 15)				
Calcium, g/100 g	1.82 ± 0.96	1.83	3.15 [2.50]	0.12 [0.50]
Phosphorus, g/100 g	1.19 ± 0.52	1.32	2.01 [1.60]	0.31 [0.40]
Ca/P ratio	1.43 ± 0.49	1.57	1.97 [2:1]	0.38 [1:1]
Cat food (*n* = 5)				
Calcium, g/100 g	1.30 ± 0.22	1.23	1.73	1.10 [0.60]
Phosphorus, g/100 g	0.95 ± 0.08	0.92	1.11	0.98 [0.50]
Ca/P ratio	1.38 ± 0.29	1.28	1.94	1.12

* Values in square brackets represent concentrations recommended by AAFCO [39] for each animal species and nutrient.

**Table 7:** Quality and safety analysis for samples per zone obtained from two pet food plants.

Establishment	Sampling Zone (*n*)	Samples above Regulatory Threshold, Microbiological Assays *					
		*Salmonella* spp.	*E. coli*	Total Coliforms	Bacterial Counts	Mold Counts	Yeast Counts
A	After mixing (5)	1	2	5	5	4	1
A	After extrusion (8)	0	0	0	0	0	0
B		0	0	0	0	0	0
A	Finished product (12)	0	0	1 ^a^	4 ^b^	1 ^c^	0
B		0	0	0 ^a^	0 ^b^	0 ^c^	0
TOTAL (25)		1	2	6	9	5	1
Reference		Legislative standards					
[30,66,67,68,69].		Absence	Absence	10–300 CFU g^−1^	5 × 10^4^ CFU g^−1^	5 × 10^3^ CFU g^−1^	5 × 10^3^ CFU g^−1^

* The sternest regulation standards are used to establish conformity criteria. Values with same letter code in the same column do not vary significantly: a (*p* = 0.152), b (*p* = 0.189), c (*p* = 0.255).

**Table 8:** Heavy metal analysis of pet foods.

Samples None Detected for Heavy Metals, *n*	Incidence, %	Concentration, μg kg^−1^			
		Mean ± SD	Median	Max	Min
Puppy food (*n* = 15)					
Arsenic					
1	93.3	20.72 ± 15.80	17.78	67.99	4.85
Cadmium					
0	100.0	89.47 ± 53.38	81.20	218.21	28.3
Mercury					
0	100.0	134.59 ± 63.44	121.36	303.47	77.11
Lead					
0	100.0	508.52 ± 305.72	375.83	1370	212.69
Adult dog food (*n* = 15)					
Cadmium					
12	20.0	13.67 ± 7.71	13.87	21.39	5.96
Arsenic					
7	46.7	10.51 ± 7.35	12.31	21.03	0.57
Mercury					
0	100.0	89.66 ± 49.78	69.31	206.94	45.64
Lead					
0	100.0	703.01 ± 705.40	562.38	2737.17	166.00

**Table 9:** Retrospective data compiled from routine inspection of puppy foods (*n* = 68) collected from 2012–2018.

Nutrient/Parameter	Mean ± SD	Median	Maximum		Minimum
Proximate analysis					
Crude protein, g/100 g	23.70 ± 5.71	25.51	34.53		9.24
Dry matter, g/100 g	91.66 ± 1.25	91.56	88.03		95.05
Crude Fiber, g/100 g	2.76 ± 1.09	2.50	6.95		0.97
Crude fat, g/100 g	13.53 ± 2.70	13.54	24.66		8.08
Crude Ash, g/100 g	10.39 ± 3.87	9.11	17.79		3.52
Nitrogen free extract, g/100 g	42.61 ± 6.93	42.27	56.25		29.57
Ethanol soluble carbohydrates, g kg^−1^	41.88 ± 7.52	43.28	58.87		30.36
Gross energy, kcal kg^−1^	4480 ± 230	4477	4935		4164
Metabolizable energy *, kcal kg^−1^	3470.9	3523.2	5273.4		2045.15
		Mineral profile			
Calcium, g/100 g	2.47 ± 1.27	2.06	5.28		0.22
Phosphorus, g/100 g	1.60 ± 0.75	1.41	4.19		0.48
Ca/P ratio	1.38 ± 0.35	1.51	1.89		0.75
Iron, mg kg^−1^	274.02 ± 105.00	247.55	500.49		115.14
Selenium, mg kg^−1^	2.13 ± 1.80	1.19	5.82		0.21
Microbiological analysis					
Indicator	Accepted minimum parameters		Number of positive samples or above limit of detection [samples above legal threshold], *n*		Prevalence, %
Total aerobic mesophilic bacterial count, CFU g^−1^	5 × 10^4^		29 [0]		42.86
Yeasts, CFU g^−1^	5 × 10^3^		2 [0]		2.94
Molds, CFU g^−1^	5 × 10^3^		1 [0]		1.47
Total coliforms, MPN g^−1^	1 × 10^3^		48 [0]		71.43
*Escherichia coli*, MPN g^−1^	Absence in 10 g		0 [0]		0.00
*Salmonella* spp.	Absence in 25 g		1 [1]		1.47
**Indicator/Parameter**	**Mean ± SD**	**Median**	**Maximum**		**Minimum**
Total counts, CFU g^−1^	382 ± 340	158	1000		100
Total coliforms, MPN g^−1^	62.80 ± 47.57	43	150		23
Mycotoxins					
Toxin/Parameter	Mean ± SD	Median	Maximum	Minimum	Prevalence, % [number of samples > limit of detection]
Aflatoxin B_1_, µg kg^−1^	0.55 ± 0.20	0.54	0.80	0.31	4.41 [*n* = 3]
Deoxynivalenol, mg kg^−1^	0.94 ± 1.22	0.47	3.92	0.35	21.43 [*n* = 15]
Fumonisin B_1_, mg kg^−1^	5.54 ± 5.42	4.33	18.91	0.06	45.25 [*n* = 31]

* Values calculated according to the European Pet Food Industry Federation (FEDIAF) [35].

**Table 10:** Retrospective data compiled from routine inspection of adult dog foods (*n* = 158) collected from 2012–2018.

Nutrient/Parameter	Mean ± SD	Median	Maximum	Minimum
Proximate analysis				
Crude protein, g/100 g	20.31 ± 4.65	19.61	34.83	10.31
Dry matter, g/100 g	91.32 ± 1.38	91.23	87.96	95.41
Crude Fiber, g/100 g	3.27 ± 1.16	3.16	7.30	1.18
Crude fat, g/100 g	12.91 ± 2.52	12.69	20.19	6.08
Crude Ash, g/100 g	8.39 ± 1.86	8.22	13.06	3.95
Nitrogen free extract, g/100 g	46.90 ± 5.16	47.05	60.24	30.96
Metabolizable energy *, kcal kg^−1^	3449.7	3411.75	5043.6	1961.25
Mineral profile				
Calcium, g/100 g	1.91 ± 0.73	1.89	3.65	0.49
Phosphorus, g/100 g	1.25 ± 0.40	1.31	2.33	0.52
Ca/P ratio	1.53 ± 0.32	1.51	3.16	0.74
Microbiological analysis				
Indicator	Accepted minimum parameters		Number of samples, *n*	Prevalence, %
*Salmonella* spp. (presence in 25 g)	Absence in 25 g		*n* = 2/158	1.26

* Values calculated according to FEDIAF [35].

**Table 11:** Retrospective data compiled from routine inspection of cat food (*n* = 25) collected from 2012–2018.

Nutrient/Parameter	Mean ± SD	Median	Maximum	Minimum
Proximate analysis				
Crude protein, g/100 g	29.00 ± 4.58	29.71	39.36	18.46
Dry matter, g/100 g	92.39 ± 0.91	92.46	94.18	90.87
Crude Fiber, g/100 g	2.30 ± 0.74	2.03	4.60	1.59
Crude fat, g/100 g	12.26 ± 3.25	12.57	19.24	5.55
Crude Ash, g/100 g	9.27 ± 2.27	8.51	14.47	5.42
Nitrogen free extract, g/100 g	39.04 ± 6.17	39.85	50.16	27.84
Metabolizable energy *, kcal kg^−1^	3423.5	3503.05	4768.6	2092.25
Mineral profile				
Calcium, g/100 g	1.72 ± 0.80	1.45	3.75	0.74
Phosphorus, g/100 g	1.43 ± 0.60	1.38	2.78	0.76
Ca/P ratio	1.20 ± 0.18	1.20	1.49	0.76
Microbiological analysis				
Indicator	Accepted minimum parameters		Number of samples, *n*	Prevalence, %
*Salmonella* spp. (presence in 25 g)	Absence in 25 g		*n* = 1/25	4.00

* Values calculated according to FEDIAF [35].

## Appendix A

**Table A1:** RTCA 65.05.63:11 criteria for evaluating feed processing plants and their severity.

**2. Documentation**
2.1 Legal permits to date [CR]
2.2 A valid, approved, updated GMP manual [MJ]
2.3 Updated records [MJ]
2.4 Records for the control of process parameters for production equipment (e.g., temperature, moisture, pressure) [MJ]
2.5 A clearly defined and documented training program [MJ]
2.6 In written, management chain and personnel responsibilities for the attention of official controls and quality assurance and safety [MJ]
2.7 Production process flow chart [MN]
2.8 Procedure or protocol for ingredient addition [MJ]
2.9 Records of internal verifications, links or officers, and corrective actions are taken [MJ]
2.10 Dry, wet, chemical and/or heat cleaning procedures, or the use of bleaching or flushing, when necessary [MJ]
**3. Facilities**
3.1 Located at distances that do not involve risks against product safety, animal health, public, and environment [CR]
3.2 The factory design minimizes the risks of manufacturing errors, allows quality control, hygiene and work safety activities [MJ]
3.3 There is sufficient or adequate space for equipment installation and production, hygiene and cleaning operations, equipment maintenance, inspection and corrective measures [MJ]
3.4 There are adequate access conditions for people and light and heavy vehicles that comply with the biosafety standards established by the company [MJ]
3.5 It has areas for handling rejection, retention or quarantine products [MJ]
3.6 Distribution areas are defined according to the GMP Regulation [MJ]
3.7 It has separate areas for handling and storing hazardous substances: pesticides, explosive materials and others [CR]
3.8 It has a separate boiler area, when applicable [MN]
3.9 The surroundings, accesses, and drains have adequate and clean maintenance so that they do not constitute sources of contamination or obstacles for emergency actions [MN]
3.10 Ceilings, floors, walls, windows and doors are designed appropriately to facilitate cleaning and disinfection; avoiding the entry and proliferation of pests [MJ]
3.11 The buildings have adequate ventilation and lighting systems for each area and operation and following current regulations [MN]
3.12 It has bathrooms, toilets, sink, rest area, dining room, and dressing rooms according to the number of people, separated from the production and storage areas [MJ]
3.13 It has systems for regular access of people and vehicles to the facilities, as well as disinfection [MJ]
3.14 Metal materials, construction, and tools are maintained in specific areas and external to the production flow or, if applicable, in safe cabinets [MN]
3.15 All work surfaces that are in contact with food allow effective cleaning and disinfection and do not represent a potential contamination factor [MJ]
3.16 It has enough garbage dumps, and they have their respective lid and identification [MN]
3.17 There is a maintenance and hygiene program of the facilities and equipment that includes the Standardized Sanitation Operating Procedures, when required [MJ]
3.18 The pallets in the process and storage areas are maintained clean [MN]
3.19 There is a specific area for storage of the finished product, and it meets the storage conditions [MJ]
**4. Equipment**
4.1 Production equipment is included and used in such a way that they have no source of contamination for food [MJ]
4.2 Magnets and screens are routinely checked to ensure proper operation and cleaning [MJ]
4.3 The measuring devices must be appropriate for the determination of the weights and/or volumes to be measured and guarantee operation through a preventive and corrective program of constant maintenance and calibration [MJ]
4.4 The performance of the mixers is checked to determine the quality of homogenization [MJ]
4.5 Pre-mixers, mixers, pelletizers and extruders are used according to the established capacity of the equipment and the manufacturer’s specifications [MJ]
4.6 A equipment cleaning and hygiene program is implemented [MJ]
4.7 The equipment is cleaned at each formula change, as in the case of single-line systems, where risk ingredients are used [CR]
**5. Personnel**
5.1 The staff is trained according to their responsibilities, and a training program for all staff is adequately implemented [MJ]
5.2 Personnel hygiene standards are defined and implemented [MJ]
5.3 Company personnel are subject to periodic health checks and records are maintained, and appropriate safety equipment is used [MN]
**6. Pest Control**
6.1 A Pest Control Program exists and is active [MJ]
6.2 There is an updated diagram of the location of the pest traps [MN]
6.3 Trained personnel correctly implement the pest control program, when applicable [MN]
6.4 Rodent control in the production area during the process and storage of finished product is adequate [MJ]
**PRODUCTION PROCESS**
**7. Production flow**
7.1 It allows continuity from the reception of ingredients to the output of the final product [MJ]
**8. Raw materials**
8.1 There is a properly documented provider registry [MN]
8.2 Raw materials, when applicable, are subject to a quarantine period and in an exclusive area for that purpose [MJ]
8.3 Raw materials are stored in specific and identified areas and separated from process areas and contaminants [MJ]
8.4 The inventory program is met “first to enter, first to leave” or “first to expire first to leave" [MN]
8.5 There is control of raw materials that do not allow the use of expired and/or contaminated ingredients [MJ]
8.6 Raw material storage areas allow inspection, cleaning, and aeration [MN]
8.7 Reused bags are subjected to disinfection processes approved by the Competent Authority [MJ]
**9. Storage of Risk Ingredients**
9.1 Raw materials that are risk ingredients are handled according to existing regulations [CR]
9.2 The areas for storage of medicines and ingredients of risk have the required conditions [MJ]
**10. Water**
10.1 There are procedures for the use and control of water quality and monitoring records are maintained [MJ]
**ELABORATION PROCESS**
**11. Formulation**
11.1 There is a procedure for the verification of formulas by competent personnel [MJ]
11.2 The formulas contain complete information and all the precautions required for the handling and use of risky ingredients [MJ]
**12. Grinding**
12.1 Constant control of the grinding process is carried out to verify the recommended particle size for each species is achieved [MN]
**13. Adding ingredients**
13.1 A preliminary preparation process for medicines and additives premixes is executed to allow correct homogenization [MJ]
13.2 Equipment allows a homogeneous mixture for the addition of liquid materials and irrigation products [MJ]
**14. Mixing**
14.1 The mixing time is technically determined and known by the operators. Besides, regular checks are performed [MN]
**15. Packaging and Labeling**
15.1 It complies with current regulations on labeling [MJ]
15.2 The packaging complies with the provisions of the GMP Regulation [MJ]
15.3 The labels are kept organized and follow adequate inventory management, with well-defined handling and use procedures [MJ]
**16. Storage**
16.1 Finished products that contain risky ingredients must be stored in separate, identified areas, and under adequate conditions [MJ]
16.2 Proper management of finished product inventories are maintained [MN]
16.3 Finished product control does not allow the use and commercialization of expired or contaminated merchandise [MJ]
**17. Reprocessing**
17.1 Any product rejected or returned for reprocessing is identified and stored in specific areas for that purpose [MJ]
**18. Dispatch, distribution, and transportation**
18.1 The expedition and delivery is made under dispatch orders with clear information [MN]
18.2 Vehicles for distribution are inspected to ensure proper cleaning conditions for product handling [MN]
18.3 Transportation cleaning activities are carried out [MJ]
**19. Quality and safety controls**
19.1 The proper procedures for quality control of raw materials other inputs of the process are implemented [MJ]
19.2 A quality control program for the finished product, which complies with current regulations, is implemented [MJ]
19.3 Records of the established quality and safety control program are properly preserved [MJ]
19.4 Samples are protected and identified concerning the lot number from which it originated [MJ]
**POST-PROCESSING**
**20. Traceability**
20.1 It has adequate identification systems and records to allow traceability in the process chain [MJ]
20.2 It has the proper procedures to handle claims, returns and product recall [MJ]
**21. Verification of GMP**
21.1 The company has an adequate program of internal audits to keep the system under control and verify compliance with the minimum sanitary and GMP requirements [MJ]
21.2 The recommendations issued in official inspections regarding GMP are met, and the required records are maintained [MJ]
**22. Environment**
22.1 The company has solid and liquid waste management systems [MJ]
